# Genetic Background and Gene Essentiality

**DOI:** 10.3390/genes16050570

**Published:** 2025-05-13

**Authors:** Paulina Gąsienica, Katarzyna Toch, Kamila Stefania Zając-Garlacz, Marta Labocha-Derkowska

**Affiliations:** Institute of Environmental Sciences, Faculty of Biology, Jagiellonian University, 30-387 Kraków, Polandkamila2.zajac@uj.edu.pl (K.S.Z.-G.); marta_labocha@o2.pl (M.L.-D.)

**Keywords:** *Caenorhabditis elegans*, gene essentiality, RNAi knockdown, genetic background

## Abstract

Background/Objectives: Essential genes are those required for an organism’s survival and reproduction. However, gene essentiality is not absolute; it can be highly context-dependent, varying across genetic and environmental conditions. Most previous studies have assessed gene essentiality in a single genetic background, limiting our understanding of its variability. The objective of this study was to investigate how genetic background influences gene essentiality in the multicellular model organism *Caenorhabditis elegans*. Methods: We examined gene essentiality in three genetically distinct *C. elegans* strains: N2, LKC34, and MY16. A total of 294 genes were selected for RNA interference (RNAi) knockdown: 101 previously classified as essential, 175 as nonessential and 18 as conditional (condition-dependent essentiality). Each gene–strain combination was tested in multiple biological and technical replicates, and rigorous quality control and statistical analyses were used to identify strain-specific effects. Results: Our results demonstrate substantial variation in gene essentiality across genetic backgrounds. Among the 101 genes previously identified as essential in the N2 strain, only 56% were consistently essential in all three strains. We identified 23 genes that were newly essential across all strains, 13 genes essential in two strains, and 9 genes essential in only one strain. These results reveal that a significant proportion of essential genes exhibit strain-dependent essentiality. Conclusions: This study underscores the importance of genetic context in determining gene essentiality. Our findings suggest that relying on a single genetic background, such as N2, may lead to an incomplete or misleading view of gene essentiality. Understanding context-dependent gene essentiality has important implications for functional genomics, evolutionary biology, and potentially for translational research where genetic background can modulate phenotypic outcomes.

## 1. Introduction

The concept of essential genes was introduced by Gluecksohn-Waelsch in 1963. These genes are responsible for fundamental biological functions that enable an organism to survive and reproduce [1]. Understanding essential genes is crucial for gaining insights into genome function, organization, and evolution. However, several key questions remain unanswered: How many genes are essential in a given organism? What is the minimal set of genes required to sustain life? Do essential genes impose evolutionary constraints and show higher conservation? Addressing these questions is fundamental to advancing our knowledge of gene function and evolution.

One reason we still lack definitive answers about gene essentiality is that large-scale “whole-organism” essentiality studies were only initiated less than 20 years ago. Earlier efforts were limited by the incomplete knowledge of the genomes and technical constraints. Studying the essentialome—the complete set of essential genes in an organism—requires both a fully sequenced and well-annotated genome to define the set of target genes and high-throughput methods to systematically inactivate each one. The first complete genome sequences of free-living organisms, *Haemophilus influenzae* and *Mycoplasma genitalium*, became available in 1995 [2,3], but it took another four years before the first essentialome was reported for *M. genitalium* [4]. While whole-genome sequencing is now routine, with thousands of assembled and annotated genomes available, gene essentiality studies remain resource-intensive and time-consuming, leading to a lag in their progress. So far, approximately 50 essentialomes have been characterized, mostly in bacteria (DEG Database) [5].

In eukaryotes, essential gene studies are particularly challenging due to larger and more complex genomes, functional redundancy, and the presence of developmental and tissue-specific essentiality. It is therefore not surprising that estimates of the proportion of essential genes vary widely, from ~2% in the multicellular animal *Drosophila melanogaster* to ~80% in the intracellular bacterium *M. genitalium* [6]. Even within the same species, essential gene estimates can differ between studies, likely due to methodological differences. For example, in *Escherichia coli*, Gerdes et al. [7] identified 14% of genes as essential, whereas Baba et al. [8] found only 7%. While methodological variability explains some of these discrepancies, differences across species may reflect fundamental biological variation in genome structure, redundancy, and regulatory mechanisms.

A second reason why fundamental questions about gene essentiality remain unanswered is that genes do not function in isolation. Instead, they interact with other genes and with the environment. As a result, a gene that is essential in one genetic or environmental context may not necessarily be essential in another [9,10]. Gene essentiality can even vary between different cell lines of the same organism [11]. This context dependence likely contributes to the absence of a single gene that is universally conserved across all prokaryotes [12]. To fully understand the nature of essential genes, it is crucial to explore how essentiality varies across genetic and environmental conditions.

To address this, we assessed the essentiality of 294 genes—101 previously classified as essential, 175 as nonessential, and 18 as conditional (condition-dependent essentiality)—across three genetic backgrounds of the nematode *C. elegans*. Specifically, we examined three wild-type strains (N2, LKC34, and MY16) and used RNA interference (RNAi) to systematically knock down the expression of our target genes.

## 2. Materials and Methods

### 2.1. C. elegans Strains

We used three wild-type *C. elegans* strains: N2, LKC34, and MY16. All strains were obtained from the Caenorhabditis Genetics Center and stored at −80 °C upon delivery. During experiments, worms were maintained at 20 °C on standard nematode growth medium (NGM) agar plates seeded with *E. coli* OP50 as a food source [13].

The N2 strain, isolated by L.N. Staniland in 1951 in England, is the standard wild-type strain adapted to laboratory conditions and is sensitive to RNA interference (RNAi) in both somatic and embryonic cells [9]. LKC34 was isolated in 2005 in Madagascar by V. Stowell, and MY16 in 2002 in Germany by H. Schulenburg. In preliminary experiments, both LKC34 and MY16 were confirmed to be RNAi-sensitive in somatic and embryonic tissues. We assessed RNAi efficacy by feeding *C. elegans* strains with RNAi bacteria: HT115 (our control, bacteria with empty vector), *hsp-4* (somatic), *par-6*, and *klp-15* (embryonic). The methodology for this preliminary experiment was identical to that used in the main experiment and is described in detail below. Fitness differences were analyzed using Kruskal–Wallis tests. Both MY16 and LKC34 strains exhibited mutant phenotypes, as indicated by significantly reduced fitness following *klp-15* and *hsp-4* RNAi treatment compared to HT115. For LKC34, the Kruskal–Wallis test yielded χ^2^ = 73.46, *p* < 0.000; post hoc pairwise Wilcoxon tests with Bonferroni correction showed significant differences between *klp-15* vs. HT115 (*p* < 0.000) and *hsp-4* vs. HT115 (*p* < 0.000). For MY16, χ^2^ = 84.50, *p* < 0.000, with similar post hoc results (*klp-15* vs. HT115, *p* < 0.000; *hsp-4* vs. HT115, *p* < 0.000). Likewise, *par-6* RNAi resulted in significant fitness reduction compared to HT115: for LKC34, χ^2^ = 68.62, *p* < 0.000 (Bonferroni-corrected); for MY16, χ^2^ = 62.30, *p* < 0.000 (Bonferroni-corrected).

### 2.2. Query Genes

As part of a larger study, we selected genes that have 1:1 orthologs in *Caenorhabditis briggsae*. From this pool, we randomly chose 294 genes—107 essential and 187 nonessential —based on information from the OGEE (Online Gene Essentiality) database and published literature [14,15]. However, while performing the experiment, the OGEE classification changed. It resulted in 101 genes being classified as essential, 175 as nonessential and 18 as conditional, which means that the essentiality of the genes was changing depending on the genetic background.

### 2.3. RNA Interference and Bacterial Preparation

To induce gene knockdown, worms were fed RNAi bacteria producing double-stranded RNA (dsRNA) specific to the selected genes [16]. RNAi clones of *E. coli* were sourced from the Ahringer RNAi library (Source BioScience, Nottingham, United Kingdom). A full list of clones is provided in Supplementary Table S1.

RNAi bacterial clones were inoculated in 5 mL of LB medium containing ampicillin (final concentration 50 µg/mL) and incubated at 37 °C with shaking for 15 h. Expression of dsRNA was induced by adding IPTG (final concentration 953 µg/mL), followed by an additional 1 h incubation under the same conditions. Cultures were then centrifuged at 3700 rpm for 40 min, supernatant discarded, and pellets resuspended in 900 µL of S Medium [13] supplemented with ampicillin and IPTG (final concentrations 100 µg/mL and 953 µg/mL, respectively).

Optical density (OD) at 600 nm was measured using a Sunrise Tecan plate reader (70 µL volume: 50 µL resuspended bacteria + 20 µL S Medium). The bacterial suspension was diluted to an OD of 0.85. Experimental 96-well plates were prepared with 50 µL of bacterial solution per well.

### 2.4. Worm Preparation

Gravid adults were bleached using standard NaOH/sodium hypochlorite methods [13], and eggs were transferred to unseeded NGM plates to hatch overnight. Starved L1 larvae were collected the next day, washed with S Medium containing ampicillin and IPTG (final concentrations 100 µg/mL and 953 µg/mL, respectively), and dispensed (20 µL per well) into 96-well plates containing RNAi bacteria. Each well contained approximately 20 worms in a total volume of 70 µL. Worms from strains used in this study had similar growth rates; hence, they did not require any adjustments of bacterial concentration.

### 2.5. Fitness Assay

An initial OD reading was taken immediately after worm addition, with a second reading taken five days later. Prior to each reading, plates were shaken for 2 min. Fitness was inferred from the amount of bacteria consumed, following the approach of Elvin et al. [17].

Each RNAi bacteria/strain combination (referred to as a “mutant” throughout the text) was tested in at least 32 replicates: two biological replicates (conducted on separate dates), each with 16 technical replicates (wells). Controls, in which worms were fed HT115 (DE3) bacteria carrying an empty vector, were included in each run with the same level of replication.

### 2.6. Assay Quality Control

We excluded wells that did not meet the following criteria:

(i) Initial OD between 0.745 and 0.905;
(ii) Worm density between 10 and 30 individuals per well;
(iii) Absence of contamination with bacteria or fungi, as assessed visually.

Preliminary testing indicated that low bacterial concentrations failed to sustain worm growth, while overly dense cultures led to increased mortality.

### 2.7. Data Standardization

Each well’s final OD (day 5) was standardized to its initial OD (day 1), which was normalized to 1. The difference between these readings served as a proxy for bacterial consumption—and hence, worm fitness. Each mutant’s fitness value was further normalized (divided by) to the corresponding wild-type strain’s (*C. elegans* strain fed with HT115 bacteria with empty vector) mean fitness from the same experimental run. These normalized values are referred to as “fitness” throughout the manuscript. Hence, the more bacteria were eaten, the higher the fitness was.

### 2.8. Data Quality Control

Step 1: For each strain, the coefficient of variation (CV =Standard Deviation/Mean) of fitness values was calculated from all measurements per run. If the CV exceeded 10%, the most deviant data point was excluded iteratively until the CV fell below this threshold. Additionally, if the range (maximum–minimum) exceeded 0.5, outliers furthest from the mean were excluded until the range was ≤0.5. The resulting data were used to calculate the wild-type strain’s mean fitness for each run.

Step 2: All mutant fitness values from the same strain and run were divided by this mean wild-type fitness.

Step 3: Standardized mutant data from different runs were combined. If the CV across runs exceeded 20%, outliers were excluded iteratively until the CV was below 20%. Similarly, if the range exceeded 0.5, extreme values were removed. Mutants with fewer than 10 remaining replicates after this filtering were excluded from further analysis. All quality control thresholds were based on empirical data.

### 2.9. Statistical Analysis

All statistical analyses were conducted in R. As the data were non-normally distributed, even after transformation, we employed a nonparametric analysis of variance using the Aligned Rank Transform (ARTool) R package (4.4.2) to test for strain (genetic background)-by-gene (RNAi) interaction.

To assess whether each mutant had significantly reduced fitness compared to its corresponding wild-type strain, we used a one-sided *t*-test (for mutants with ≥30 data points) or a Wilcoxon test (for mutants with <30 data points). Resulting *p*-values were adjusted for multiple testing using the false discovery rate (FDR) method [18].

### 2.10. Gene Ontology

Gene function was annotated using the Gene Ontology (GO) database [19] via the BioMart tool integrated with WormBase (release WS290) [20].

## 3. Results

The final dataset included 294 genes. A nonparametric analysis of variance revealed that both genetic background (strain) and gene knockdown (RNAi), as well as their interaction, had a significant effect on *C. elegans* fitness (Table 1).

To evaluate consistency with previous annotations, we compared our experimental classification of genes (essential vs. nonessential) in the N2 strain to classifications in the OGEE database, which is also based on the N2 background. We applied three criteria to define essentiality, one based on statistical significance, and two based on fitness thresholds as proposed by Ramani et al. [21]:

1. Statistical significance: Genes were classified as essential if their fitness was significantly lower than that of wild-type worms (adjusted *p* < 0.01 from a *t*-test or Wilcoxon test). This method showed 58% agreement with OGEE (Supplementary Table S1).
2. Fitness threshold I: Genes with an average fitness <0.9 of wild-type fitness were considered essential. This method yielded 68% agreement with OGEE (Supplementary Table S1).
3. Fitness threshold II: Genes with an average fitness <0.75 of wild-type fitness were considered essential. This criterion produced the highest agreement with OGEE at 70%. We adopted this method for all subsequent analyses.

Using this 0.75 fitness threshold, we found that of the 101 genes classified as essential in OGEE, 62 were essential in the N2 strain, 59 in LKC34, and 72 in MY16. In total, 57 OGEE-essential genes were essential across all three strains, and an additional 15 were essential in at least one strain (Supplementary Table S1).

**Figure 1:**
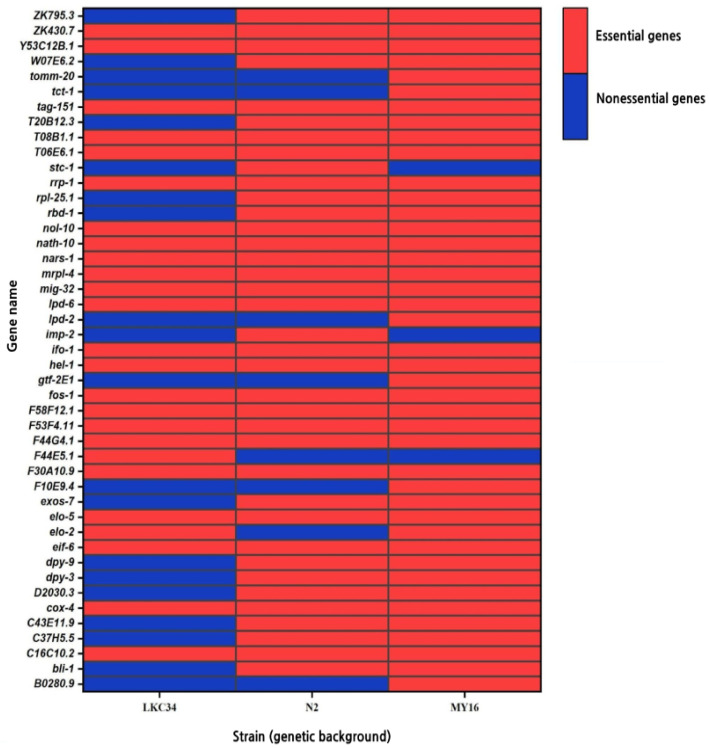
Genes classified as essential in our experiment that were previously classified as nonessential.

Among the 175 genes labeled as nonessential in OGEE, 143 remained nonessential in N2, 154 in LKC34, and 138 in MY16. However, 45 OGEE-nonessential genes (also considering those that were now classified as conditional, whereas previously they were classified as nonessential) were essential in at least one genetic background in our study (Figure 1, Supplementary Table S1).

**Figure 2:**
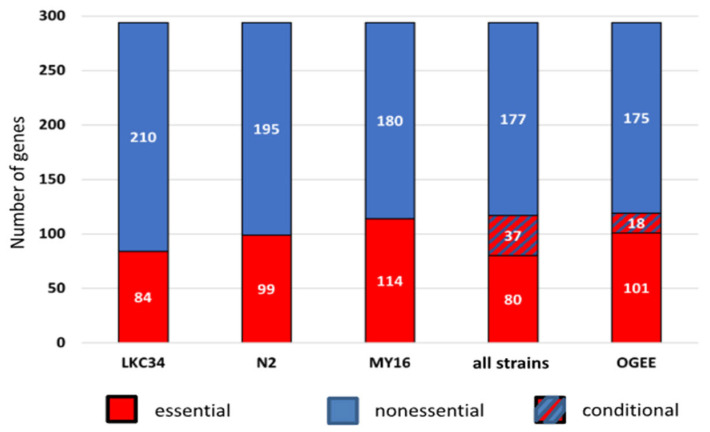
Number of genes classified as essential, nonessential, and conditional (essential/nonessential depending on the strain). Data for three different genetic backgrounds (strains: LKC34, N2, and MY16) are presented separately and jointly (all strains). OGEE—classification according to the OGEE database.

**Figure 3:**
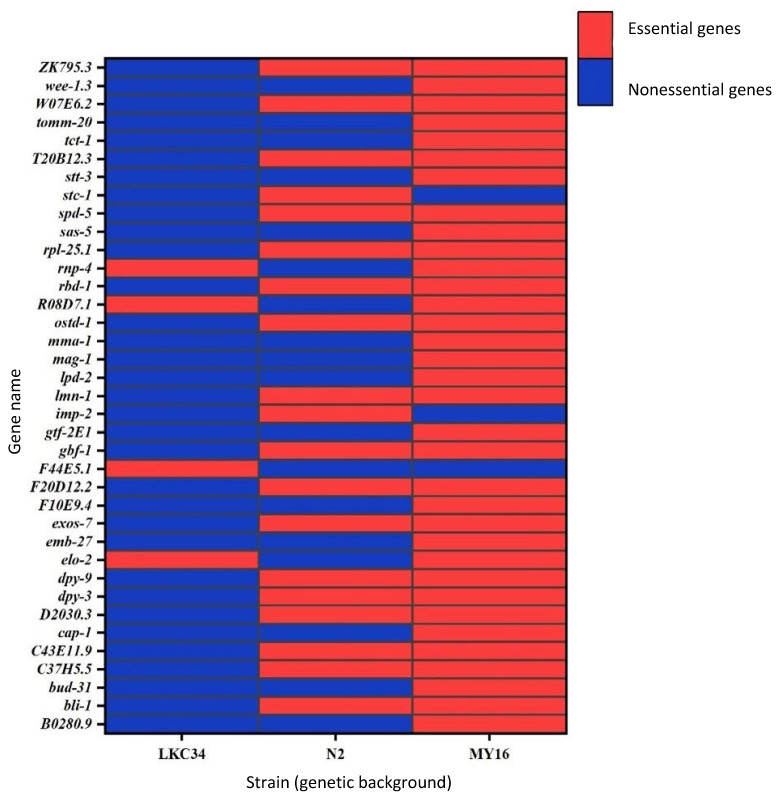
Genes for which differences in classification (essential/nonessential) were observed between genetic backgrounds (strains).

Overall, 80 genes were essential in all three strains, while 177 were nonessential across all backgrounds (Figure 2, Supplementary Table S1). For 37 genes, classification as essential or nonessential varied depending on the genetic background (Figure 3, Supplementary Table S1).

**Figure 4:**
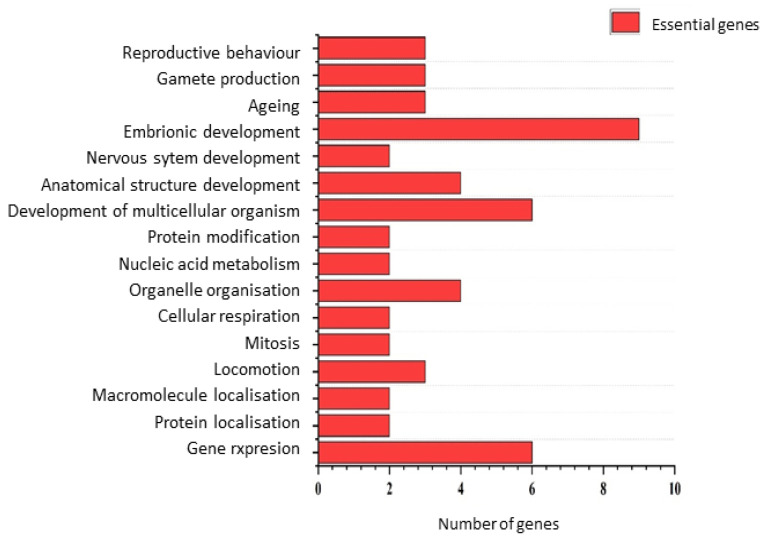
Gene Ontology biological process categories of genes classified as essential in all three strains. Only functions represented by at least two genes are shown.

**Figure 5:**
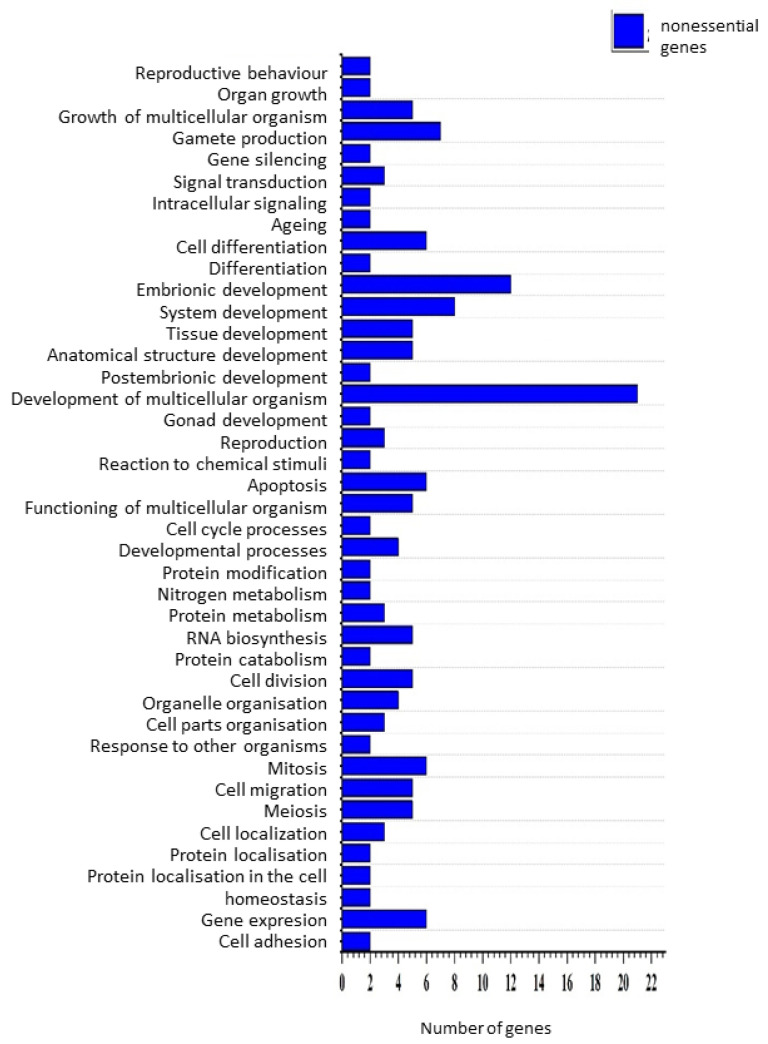
Gene Ontology biological process categories of genes classified as nonessential in all three strains. Only functions represented by at least two genes are shown.

Gene Ontology (GO) analysis showed that essential genes were enriched for terms related to embryonic development, whereas nonessential genes were more commonly associated with processes involved in multicellular organism development (Figure 4 and Figure 5).

## 4. Discussion

Gene essentiality is not a fixed attribute but a context-dependent property influenced by multiple biological factors, including genetic background. Previous studies have demonstrated that mutations in the same gene can produce divergent phenotypic outcomes across individuals of the same species [22]. One of the primary drivers of this variability is the underlying genetic architecture—often referred to as the genetic background—upon which mutations arise and act.

Our results provide strong empirical support for the hypothesis that gene essentiality can vary substantially across genetic backgrounds. We observed significant genotype-by-gene interactions: of the 175 genes previously classified as nonessential, 45 were found to be essential in at least one of the three wild-type *C. elegans* strains we tested. Interestingly, MY16—known to be more genetically distant from the standard N2 reference strain than LKC34 [23]—accounted for six of these reclassified genes, compared to only one in each of the other two strains. This suggests that increasing genetic divergence enhances the likelihood of uncovering background-dependent essentiality.

This pattern aligns with the concept of epistasis, where the phenotypic effect of a mutation depends on interactions with other loci. Such interactions are increasingly recognized as pervasive and central to the variability of phenotypic traits [24,25]. In our study, 27% of all query genes were essential across all three backgrounds. However, when conditional essentiality—genes essential in only one or two strains—was considered, this number increased to 40%. These findings indicate that each additional genetic background assessed contributes new insights, both in terms of newly identified essential genes and in revealing previously essential genes as nonessential under different genetic contexts.

While some discrepancies in essentiality might be attributed to experimental or environmental differences—particularly when comparing to prior foundational studies such as Kamath et al. [14], on which classification of genes in the OGEE is based—we controlled for many variables by using standardized laboratory conditions. Moreover, although we included the N2 strain, its genetic profile may have diverged slightly from the one used two decades ago, given the documented accumulation of spontaneous mutations over laboratory time [26].

Gene Ontology (GO) analysis offered additional insights. Essential genes in our dataset occurred in categories related to embryonic development, multicellular organismal development, gene expression, and organelle organization—findings consistent with previous works suggesting these biological processes are particularly sensitive to perturbation [27,28]. Notably, nonessential genes were also associated with embryonic and developmental processes, reinforcing the idea that gene essentiality does not necessarily reflect biological unimportance. Rather, redundancy and compensatory pathways may allow certain critical processes to be buffered against loss-of-function mutations in specific backgrounds [29,30].

Other studies have similarly reported background-dependent variation in gene function, particularly in early development [9,22]. Our findings add to this growing body of evidence and emphasize the need to reframe essentiality as a conditional rather than absolute trait.

The search for a universal set of essential genes—or the so-called “essentialome”—remains a key objective in genetics and systems biology. Such data could drive progress in fields as diverse as minimal genome design, synthetic biology, and precision medicine [12,31]. What is more, knowledge regarding essential genes could potentially serve as a resource in targeted antibacterial treatments [32,33] and cancer therapies [11,34]. However, our findings illustrate the inherent limitations of studying gene essentiality in a single reference genome or strain. Essentiality, like many other genetic properties, emerges from a dynamic interplay between genes and their contexts.

Ultimately, achieving a comprehensive understanding of gene essentiality will require systematic investigations across a broad spectrum of genetic backgrounds. This approach promises not only a more complete essentialome but also deeper insight into the principles governing genetic robustness, redundancy, and adaptability.

## Figures and Tables

**Table 1:** The effect of strain (genetic background) and mutation (RNAi) and their interaction on worm fitness; the table presents the results of the Aligned Rank Transform ANOVA from ARTool.

Variable	df	F-Value	*p*-Value
Strain	2	3547.2	<0.001
Mutation	293	469.8	<0.001
Strain × Mutation	586	34.3	<0.001

## Data Availability

The original contributions presented in this study are included in the article/Supplementary Material. Further inquiries can be directed to the corresponding authors.

## Supplementary Materials

The following supporting information can be downloaded at https://www.mdpi.com/article/10.3390/genes16050570/s1: Table S1: RNAi clones used in the study; information about the essentiality of all genes tested in the study in each *C. elegans* strain.
